# Osteopathic Manipulative Therapy Induces Early Plasma Cytokine Release and Mobilization of a Population of Blood Dendritic Cells

**DOI:** 10.1371/journal.pone.0090132

**Published:** 2014-03-10

**Authors:** Stevan Walkowski, Manindra Singh, Juan Puertas, Michelle Pate, Kenneth Goodrum, Fabian Benencia

**Affiliations:** 1 Osteopathic Manipulative Medicine Department, Heritage College of Osteopathic Medicine, Ohio University, Athens, Ohio, United States of America; 2 Molecular and Cellular Biology Program, Ohio University, Athens, Ohio, United States of America; 3 Department of Biomedical Sciences, Heritage College of Osteopathic Medicine, Ohio University, Athens, Ohio, United States of America; 4 Biomedical Engineering Program, Russ College of Engineering and Technology, Ohio University, Athens, Ohio, United States of America; 5 Diabetes Institute, Ohio University, Athens, Ohio, United States of America; New York University, United States of America

## Abstract

It has been claimed that osteopathic manipulative treatment (OMT) is able to enhance the immune response of individuals. In particular, it has been reported that OMT has the capability to increase antibody titers, enhance the efficacy of vaccination, and upregulate the numbers of circulating leukocytes. Recently, it has been shown in human patients suffering chronic low back pain, that OMT is able to modify the levels of cytokines such as IL-6 and TNF-α in blood upon repeated treatment. Further, experimental animal models show that lymphatic pump techniques can induce a transient increase of cytokines in the lymphatic circulation. Taking into account all these data, we decided to investigate in healthy individuals the capacity of OMT to induce a rapid modification of the levels of cytokines and leukocytes in circulation. Human volunteers were subjected to a mixture of lymphatic and thoracic OMT, and shortly after the levels of several cytokines were evaluated by protein array technology and ELISA multiplex analysis, while the profile and activation status of circulating leukocytes was extensively evaluated by multicolor flow cytometry. In addition, the levels of nitric oxide and C-reactive protein (CRP) in plasma were determined. In this study, our results show that OMT was not able to induce a rapid modification in the levels of plasma nitrites or CRP or in the proportion or activation status of central memory, effector memory or naïve CD4 and CD8 T cells. A significant decrease in the proportion of a subpopulation of blood dendritic cells was detected in OMT patients. Significant differences were also detected in the levels of immune molecules such as IL-8, MCP-1, MIP-1α and most notably, G-CSF. Thus, OMT is able to induce a rapid change in the immunological profile of particular circulating cytokines and leukocytes.

## Introduction

Osteopathic medicine is based on the premise that the primary role of the physician is to facilitate the body's inherent ability to heal itself. Osteopathic physicians view diseases as a disruption of the normal interactions of anatomy, physiology and behavior. One unique aspect of osteopathic medicine is treatment by manually applied procedures, often referred to as manipulative therapies. These therapies have been successfully used by osteopathic physicians for more than a hundred years in order to treat dysfunctions of the neuromusculoskeletal, lymphatic, or vascular tissue. As described in Foundations for osteopathic medicine [1] some of the techniques relevant to osteopathy include: 1) soft-tissue techniques that increase muscle relaxation and circulation of body fluids; and 2) isometric and isotonic techniques that focus on restoring physiological movements and altered joint mechanisms. Manipulative therapies aimed to increase lymphatic flow, such as thoracic or abdominal lymphatic pump, have been extensively used in osteopathic medicine [2], [3]. In particular, these techniques are proposed to treat patients with asthma, edema and certain pulmonary infections since an increase in the lymphatic flow may enhance filtering and removal of fluid, inflammatory mediators, and waste products from interstitial spaces [4]. Interestingly, it has been claimed that osteopathic treatment decreased mortality rates associated with the 1918 influenza epidemic in the United States and may be relevant in the case of an avian influenza pandemic [5], [6]. Various reports of beneficial clinical responses to lymphatic pump treatments may be related to increased lymph flow [7], [8], [9], [10], [11]. In addition, it has been proposed that lymphatic and splenic pump treatments at the time of vaccination were correlated with a faster rise in antibody titers in human subjects receiving the hepatitis B vaccine [7]. In another series of studies, patients receiving thoracic pump treatments showed statistically significant improvement in their overall response against pneumonia, measured as the mean duration of oral antibiotic use [12]. On the contrary, several studies in humans have failed to show that osteopathic lymphatic techniques improve immune responses to influenza vaccination [13], [14], [15].

Of the few published studies that have examined changes in leukocyte counts following osteopathic lymphatic techniques [10], [16], [17], [18], [19], only small and inconsistent changes in total leukocyte count or mean percentage differential counts have been reported. Study designs and manipulative techniques have also been variable. In 1932 [17] and in 1934 [16] Castlio and Ferris-Swift reported post-treatment leukocyte numbers in 100 normal subjects or in 100 patients with acute infections. Both studies reported that splenic pump manipulation increased post-treatment (30 minutes) leukocyte count. The design of these studies lacked untreated control groups, and investigators may not have been blinded to sample identity during sample analysis. A re-analysis by Noll and Johnson in 2005 [18] and in 2008 [20] of the data from these early studies cast doubt on the statistical significance of the conclusion that splenic pump manipulation increased leukocyte counts (normal subjects). The re-analysis of data on subjects with infections did support claims of a modest increase in mean leukocyte count and a small decrease in mean percentage of combined eosinophils and basophils (30 and 45 minutes post treatment). Further, Mesina *et al* in 1998 [10] performed lymphatic pump techniques (pectoral traction and splenic pump) on 7 male medical students and reported a post-treatment increase (vs. 5 untreated control subjects) in the percentage of blood basophils. In contrast, a 2008 study by Rivers *et al* [19] reported no post-treatment leukocytosis or basophilia in 12 male subjects enrolled in a randomized crossover study design with 10-minute comprehensive lymphatic treatment and rest (control) protocols delivered 1 week apart. Notably, in a series of studies on acute pneumonia patients, application of the thoracic pump technique (another lymphatic pump technique) using standardized protocols reduced the duration of patients' use of oral and intravenous antibiotics and the length of hospital stays [12], [21].

In more recent years, exciting studies on the effect of OMT on immune parameters have been performed. Firstly, three publications from the Hodge lab have shown that abdominal lymphatic pump can significantly modify the leukocyte population in lymphatic circulation [22], [23], [24]. By using an animal experimental dog model, these researchers showed that OMT can exert a mechanotransduction stimulus that is capable of modifying immune parameters. In addition, a recent publication from the same lab showed that thoracic and abdominal lymphatic pump techniques were able to reduce *Streptococcus pneumoniae* colony-forming units in the lungs of rats with acute pneumonia [25]. Although no identification of the particular mechanism responsible for this effect has yet been determined, this data clearly highlights the capability of OMT to enhance protection against infection. Further, two recent research studies by Licciardone *et al.* on human patients suffering chronic low back pain showed that repeated OMT treatment was able to modify the levels of TNFα in circulation [26], [27]. Finally, a randomized, controlled clinical trial comparing a standardized lymphatic pump protocol with a light-touch protocol determined that OMT can induce a rapid and significant decrease in the platelet levels in elder patients [28].

Herewith, in this pilot study, we decided to investigate rapid responses on several human circulating leukocyte populations and cytokines. To this end, human volunteers were subjected to a combination of lymphatic, splenic and hepatic pump osteopathic treatments and blood samples were collected at different times pre and post-OMT. Then, the proportion of different leukocyte populations was determined by hematological techniques combined with thorough flow cytometry analysis. Further, the presence of several chemokines, cytokines and growth factors were determined in our samples by means of antibody array technology and multiplex ELISA analysis.

## Materials and Methods

### Ethics statement

This study was approved by the Ohio University Institutional Review Board Ethics (protocol 09F007) Committee and informed consent was obtained from all volunteers. The research process, including the collection and storage of blood, isolation of immune cells and determination of cytokine levels in plasma samples, was explained in detail to every participant. Every participant provided written informed consent.

### Participants

Healthy volunteers were enrolled for these studies following an institutional review board approved human subjects' blood collection protocol as described above. Selection of volunteers was with the help of the Clinical Research Unit of the Heritage College of Osteopathic Medicine, Ohio University. As described in other studies [7], smoking, pregnancy, recent (within 3 months) use of corticosteroids, cytotoxic drugs, or immunosuppressants, cardiovascular risk factors, liver disease, renal failure, or acute and chronic infections (including HIV) were used as exclusion criteria. Also excluded were individuals with recent history of broken or damaged ribs or upper thoracic injury. Potential participants were screened by telephone by research nurses from the Clinical Research Unit of the Heritage College of Osteopathic Medicine, Ohio University who explain the project and procedures to potential research subjects, respond to questions, and schedule potential subjects for participation.

As previously described for similar studies [28], upon arrival for the scheduled participation, prospective subjects were given the original telephone questionnaire in person as well as a study visit medical questionnaires for additional exclusion criteria. All participants received a written and verbal explanation of the nature of the study, study objectives and the procedures, including the obtaining of blood samples and the treatment protocols. A consent form explaining the nature of the studies, risks, benefits, possible discomforts and record keeping and confidentially was presented and explained to the participants by the research nurses. Consent was obtained from all participants enrolled for these studies. The demographic information for age and sex was collected.

For the first series of studies, 21 participants were recruited. In this group, two of the participants withdrew due to problems with recovering blood during the second or third venipuncture. Ten participants fell in the OMT group and 10 in the sham group. For the second group of studies a total of 36 participants were recruited. In this group, three participants withdrew before the treatment or after due to problems with recovering blood during the second or third venipuncture. Sixteen participants fell in the OMT group and 17 in the sham group.

### OMT Protocol

As previously described in detail by Jackson *et al.* (1998), the time length of OMT on each participant was 7 minutes [7]. The OMT protocol consisted of a mix of lymphatic, splenic and hepatic pump treatments. To accomplish this, each volunteer lay on his/her back on a treatment table. Then, the osteopathic physician placed his hands on both sides of the individual's anterior aspect of the chest and fingers pointing towards the individual's feet and heel of the physician's hand just below the inferior border of the clavicles. The osteopathic physician positioned his hands as above and then rhythmic compressions to the chest were applied for approximately 2 minutes. The physician then moved to the subject's left thoracic cage and applied rhythmic compressions to the lateral thoracic cage while holding the left upper extremity in approximately 60 degrees of abduction for approximately 2 minutes. Next, the physician moved to the right thoracic cage and repeated this procedure for another 2 minutes. The physician then returned to the upper thoracic cage and repeated the bilateral upper rib compression. Then, the volunteer was instructed to inhale deeply and then exhale fully and slowly. While the individual exhaled the osteopathic physician applied oscillatory compressions. At the third inhalation, the osteopathic physician instructed the volunteer to inhale deeply against the physician's resistance to the inhalation. This was repeated three times against gentle resistance to inhalation. After the third exhalation, the osteopathic physician then quickly released the resistance at the midpoint of the next forced inhalation, allowing the individual to suddenly expand the chest. The total time of this procedure was 7 minutes.

### Sham Protocol

For the sham treatment group, we used light touch to the similar region as described previously [29]. Here, the osteopathic physician placed his hands on the upper thoracic cage, left and right thoracic cage, and upper thoracic cage as in the OMT group. Thus, this group will act as a control for “light touch” variable.

### Randomization

For these studies we perform a simple random sampling. For each group of 10 participants, the clinician prepared 5 closed papers with OMT and 5 with sham labels in a closed box. At the day of the treatment, and after the participants signing the consent forms, the clinician drew one paper in order to determine to which group the participant was allocated. The participants were not informed whether they were subjected to OMT or sham treatment.

### Blinding

All outcome analyses were performed in a blinded fashion. The researchers in charge of performing the hematological and immunological techniques did not receive any information regarding whether the samples belong to an OMT or sham-treated participant until the stage of statistical analysis, and did not witness the protocol treatments.

### Outcomes

#### Blood collection

Blood was drawn by research nurses from OMT and sham-treated participants at −1 h (baseline), 5 and 30 min after treatment (first series of experiments, OMT = 10 participants, sham = 10 participants); or at −1 h, 30 min and 1 h after treatment (second series of experiments, OMT = 16 participants, sham = 17 participants). Two 8-ml VACUTAINER CPT (Cell Preparation Tube) and one 8-ml VACUTAINER PPT (Plasma Preparation Tube) (all BD, Franklin Lakes, NJ), containing citrate as the anti-coagulant, were collected from each subject. Samples were treated according to the manufacturer's instruction in order to obtain plasma for nitrite determinations and mononuclear cells for different studies.

#### Evaluation of total number of leukocytes in blood samples and differential count

Total and differential leukocyte numbers were determined in an automatic cell counter in triplicate (HemaVet HV950FS programmed for human blood, Drew Scientific, Inc., Oxford, CT). To confirm differential leukocyte counts, peripheral blood smears were made and stained with Wright Giemsa stain for each specimen. Two hundred white blood cells were counted at a 1000× magnification. Then, differences in the numbers and types of leukocytes among samples recovered from OMT and sham treated individuals were determined.

#### Evaluation of the phenotype of blood leukocytes by means of flow cytometry analysis

In order to analyze the phenotype of circulating leukocytes from OMT and sham treated participants, mononuclear cells were stained with specific fluorochrome-labeled antibodies and analyzed by flow cytometry. All samples were stained with CD45 (2D1) antibody (BD Biosciences, San Jose, CA) a pan leukocyte marker. In order to define T lymphocyte subsets, CD3 (HIT3a), and CD8 (HIT8a) antibodies were used. Further staining with CD45RA and CCR7 (3D12) helped to identify naïve and memory lymphocytes within the T cell population. CD56 (B159) was used to detect NK cells, and NKT cells were defined as cells co-expressing CD56 and CD3. The activation status of T cells, NK cells and NKT cells was defined due to the levels of expression of the early activation antigen CD69 (FN50). B cells were defined as CD45^+^MHC-II (HLA-DR)^+^ and CD19(HIB19)^+^ cells. Monocytes were detected by means of CD14 (M5E2) staining; and different monocyte subpopulations identified by their expression of CD16 (CB16). DCs were identified by staining together with CD11c (HL3, myeloid DCs) or CD123 (9F5, plasmacytoid DCs). Different myeloid DC populations were identified by their expression of CD1c (L161), CD16 (CB16) and CD141 (1A4, BD Biosciences). The activation status of circulating APCs (B cells, monocytes and DCs) was defined based on the levels of expression of HLA-DR, CD80 (2D10.4) or CD86 (IT2.2). All antibodies except where indicated were acquired from eBioscience (San Diego, CA).

#### Analysis of metabolites in plasma samples

Presence of cytokines, chemokine and growth factors in plasma samples obtained from OMT and sham participants was analyzed using a cytokine antibody array (Human Inflammatory Array; Ray Biotech, Inc., Norcross, GA) following the manufacturer's instructions. Briefly, samples were seeded on glass microarrays and upon following incubation and washing procedures fluorescence signal intensities were measured in an Agilent microarray B-scanner (Agilent Technologies, Inc.; Santa Clara, CA). Once the fluorescent intensity data was obtained, the background was subtracted and the values normalized to the provided positive control signals before proceeding to analysis. Then, comparison of signal intensities for antigen-specific antibody spots was used to determine the relative differences in expression levels of each analyte between samples. As per manufacturer's instructions, any ≥1.5-fold increase or >0.65-fold decrease in signal intensity for a single analyte between samples was considered a measurable and significant difference in expression. This assay, as followed, is not able to assess absolute or relative concentrations of different analytes in the same sample. Plasma samples (pretreatment and 30 min post-treatment) from OMT and control subjects were compared for changing levels of 40 different cytokines and chemokines associated with inflammatory responses.

In another set of experiments, plasma samples obtained from OMT and sham participants (0 min, 30 min and 1 h post-treatment) were analyzed for the presence of chemokines and cytokines by using a MILLIPLEX MAP Human Cytokine/Chemokine Panel (Millipore, Billerica, MA) according to manufacturer's specifications. The chemokines and cytokines chosen for analysis were Eotaxin, G-CSF, IL-1α, IL-1β, GM-CSF, IL-2, IL-8, IL-4, IL-6, IL-10, MCP-1, and MIP-1α.

The levels of C-reactive protein (CRP) in plasma samples were determined by using a human CRP ELISA kit (AssayPro, St. Charles, MO) following the manufacturer's instructions.

Nitrate levels in plasma samples, a measure of nitric oxide metabolism, were determined by using the Nitrate/Nitrite Fluorometric Assay Kit (Cayman Chemical Company, Ann Arbor, MI) following the manufacturer's instructions. Briefly, fluorescent emissions were evaluated in a Synergy HT microplate reader (Biotek, Winooski, VT), using a 360 excitation filter and a 420 emission filter.

### Statistical methods

For this pilot project, multiple comparisons we performed ANOVA analysis with post-analysis comparisons by the Tukey-Kramer multiple comparisons test. When samples did not follow normal distribution, non-parametric statistics were used for analysis. When comparing the levels of a particular molecule within the OMT or sham group at different time-points, we used paired-sample statistics analysis. A value of p<0.05 was considered significant. Data are expressed as mean ± SD. Data was analyzed using Graph Pad Instat software (GraphPad Software, Inc., San Diego, CA).

## Results and Discussion

The focus of our research was to analyze rapid responses to OMT. Thus, samples were obtained for analysis within an hour of treatment. This has the benefit of “homogenizing” the volunteer behavior, since during that time they were not allowed to eat food or drink and were monitored to by our research team. On the other hand, due to the short period of time after OMT, we do not expected to observe changes due to “*de novo* synthesis” of particular immune factors, but rather an effect of changes in the secretion rates of preformed or constitutively produced molecules. Likewise, differences in the percentages of activated immune cells would surely reflect mobilization of populations rather than synthesis of markers of activation during this period of time. It is important to highlight that no adverse effects were reported due to OMT in any of the participants of these studies.

### Immediate response to OMT

**Figure 1:**
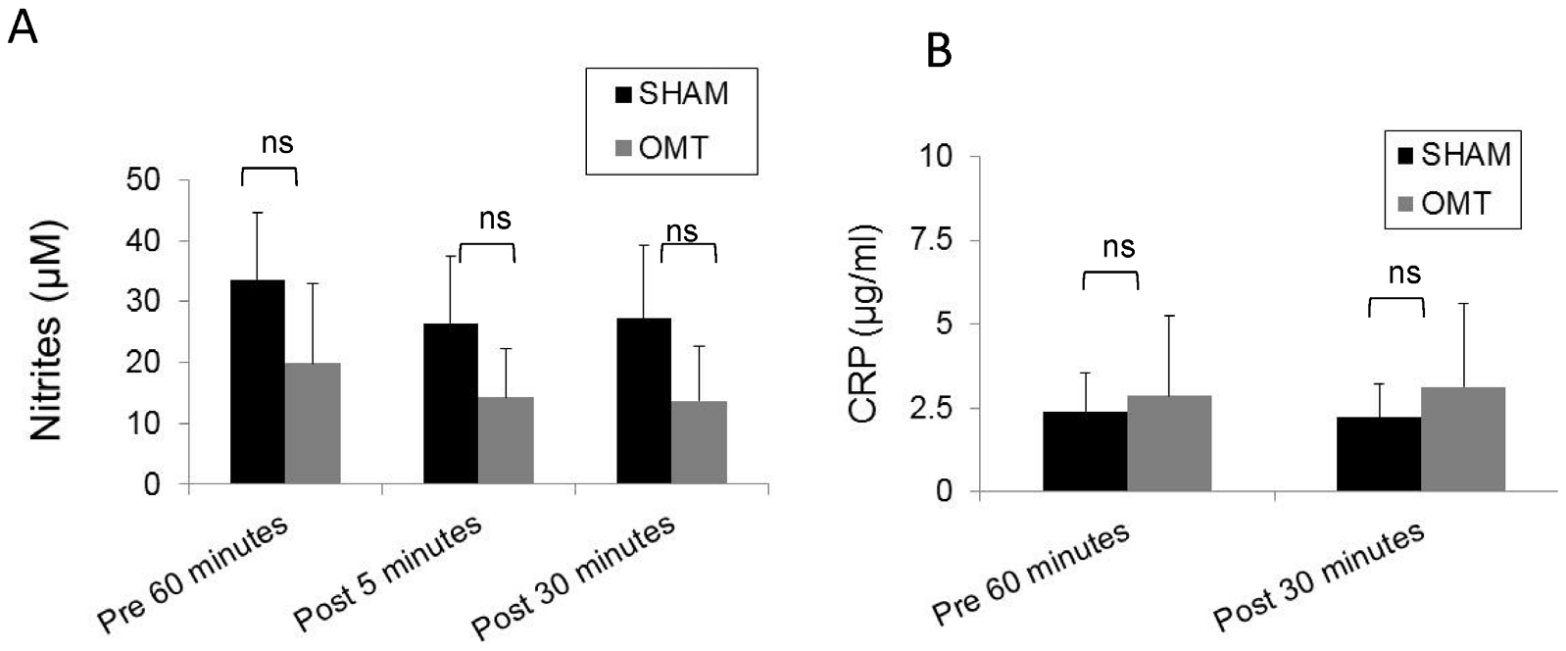
Levels of nitrites and CRP in plasma of OMT and sham experimental groups at 5 and 30-treatment. Plasma was recovered from blood samples and analyzed for the presence of nitrites (A) and CRP (B) as described in the Materials and Methods section. Data are expressed as mean ± SD. Data were analyzed by ANOVA followed by Tukey-Kramer Multiple Comparisons post-test. N = 10 OMT and 10 sham.

**Figure 2:**
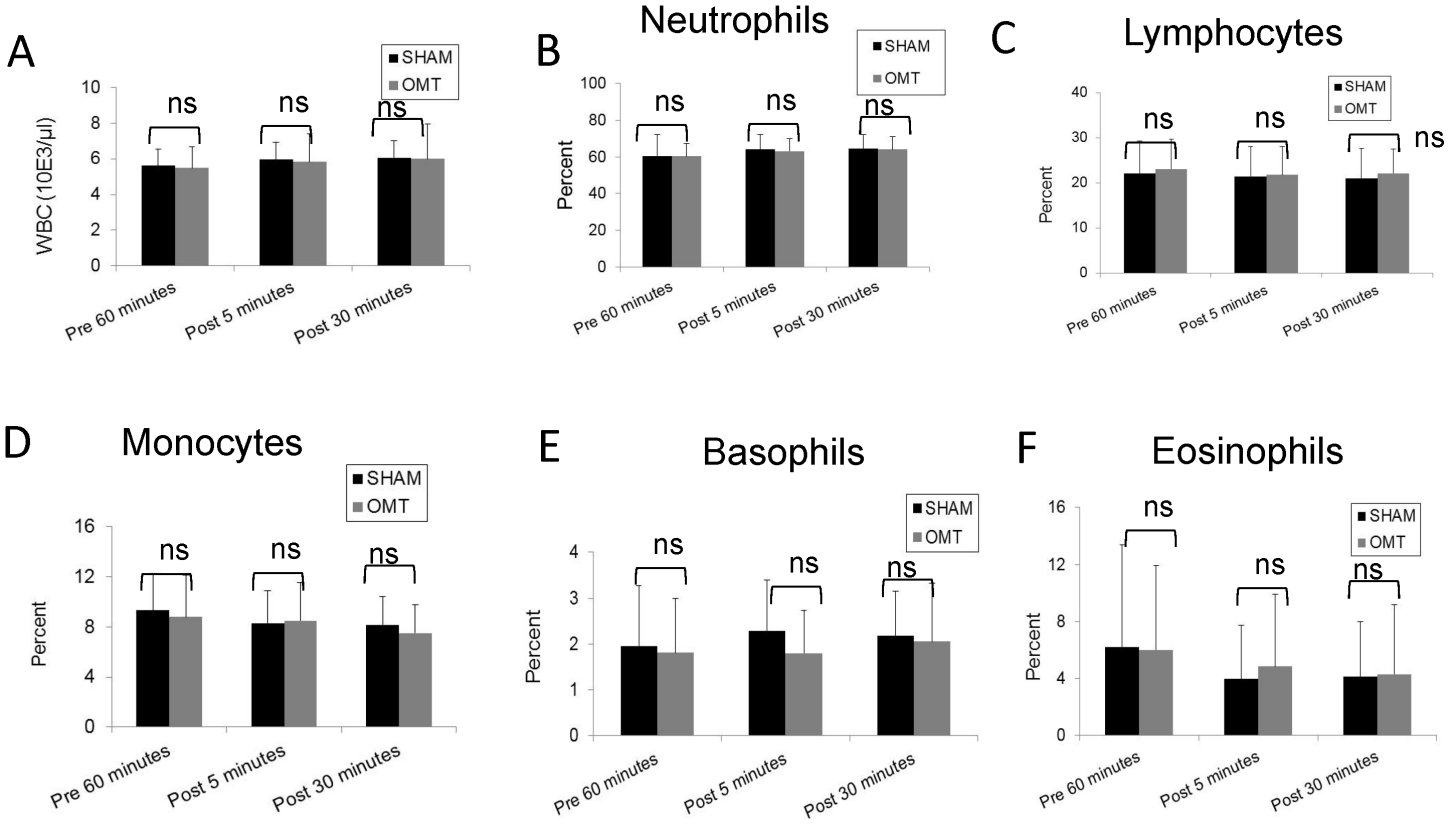
Total and differential WBC counts in OMT and sham experimental groups at 5 and 30-treatment. The total and differential numbers of white blood cells were determined by determined in an automatic cell counter in triplicate (HemaVet HV950FS programmed for human blood). Data are expressed as mean ± SD. Data were analyzed by ANOVA followed by Tukey-Kramer Multiple Comparisons post-test. N = 10 OMT and 10 sham.

**Figure 3:**
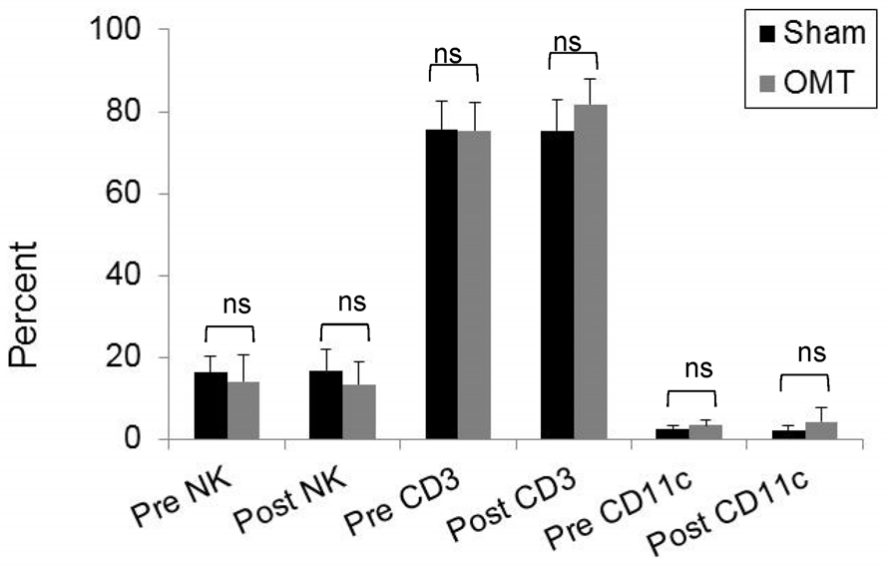
Flow cytometry analysis of circulating NK and CD3 T cell populations in OMT and sham experimental groups at 30-treatment. PBMC were obtained from blood samples and stained for CD45, NK and CD3 with fluorescent antibodies and analyzed by multicolor flow cytometer within the lymphocyte FSC vs. SSC gate. The percentage of NK and CD3 cells within the CD45 population was determined. Data are expressed as mean ± SD. Data were analyzed by ANOVA followed by Tukey-Kramer Multiple Comparisons post-test. N = 10 OMT and 10 sham.

In order to investigate the effect of OMT on the level of circulating metabolites and leukocytes, healthy volunteers were subjected to a mixture of lymphatic and hepatic pump treatments while a sham group received a “light touch” treatment. In order to determine the basal levels of circulating metabolites and leukocytes, 1 h prior treatment blood was extracted from the volunteers. To evaluate immediate response to OMT, blood was drawn at 5 and 30 min post-treatment. It has been previously reported that OMT can induce production of nitric oxide (NO) [30], but as shown in **Fig. 1A**, we were not able to detect an increase in the circulation levels of nitrites (a measure of NO metabolism) at 5 or 30 min after treatment. In addition, we investigated the levels of CRP. The levels of this acute response protein are dramatically elevated upon an inflammatory stimulus, but since its synthesized *de novo* by the liver, very important changes in the levels of this molecule are usually observed after 4 h of stimulation. Nevertheless, taking into account some reports that indicate a small but significant increase of CRP at very early time points after stimulation [31], [32], we decided to evaluate if OMT was able to induce a modification of this factor in circulation. As shown in **Fig. 1B**, no differences were observed between OMT and sham groups in the circulating levels of CRP at 5 and 30 min post-treatment. As described above, some reports indicate that OMT could be capable of modifying the levels of circulating leukocytes, but as shown in **Fig. 2**, we were not able to observe significant differences in the number or proportion of different leukocyte populations immediately after OMT. Further, flow cytometry analysis did not reveal differences in the levels of NK cells or CD3 T cells present in the PBMCs samples of OMT or sham-treated volunteers 30 min post-treatment (**Fig. 3**). These data does not contradict previous observations taking into account that we are focusing in very early responses, while other studies have focused on later responses.

In order to further investigate the rapid response of the immune system to OMT, we evaluated the levels of various chemokines, cytokines and growth factors in the plasma of OMT and sham treated subjects using antibody array technology (Human Inflammatory Array; Ray Biotech). Plasma samples (pretreatment and 30 min post-treatment) from 4 OMT and 4 control subjects were compared for changing levels of 40 different factors associated with inflammatory responses. Expression levels of four cytokines (eotaxin, eotaxin-2, IL-10, IL-16) were significantly increased (≥1.5× increase at 30 minutes vs. pretreatment level) only in OMT subjects (4 of 4 OMT subjects vs. 0 of 4 control subjects) (**Table 1**). Further, there was a general pattern of increase (≥1.5× increase) for multiple other factors in 3 of 4 OMT subjects (G-CSF, MIP-1α, sTNFR1), or 2 of 4 OMT subjects (GM-CSF, IL-1α, IL-2, IL-6, IL-8, IL-11, MCP-1, TNF-β) or vs. 0 of 4 control subjects (**Table 1**). Large variations in level of cytokine expression by individual subjects within each study group were observed with some cytokines. Similar rapid responses in the levels of cytokines have been observed in an experimental model, in which dogs subjected to lymphatic pump techniques showed a rapid discharge of cytokines in the lymphatic circulation [23]. It is noteworthy to comment that these qualitative observations on a limited number of samples suggested the possibility of a rapid change in the cytokine milieu in response to OMT.

**Table 1:** Frequency of Significant Post-treatment Increases in Cytokine Expression.

	Samples with ≥1.5× cytokine increase vs. pretreatment value			Samples with ≥1.5× cytokine increase vs. pretreatment value	
Analyte	Sham	OMT	Analyte	Sham	OMT
EOTAXIN	0	4	TIMP-2	0	0
EOTAXIN-2	0	4	IL-4	1	4
IL-10	0	4	IP-10	1	4
IL-16	0	4	I-309	1	3
GCSF	0	3	IL-12 p40	1	3
MIP-1α	0	3	IL-12 p70	1	3
s TNF RI	0	3	IL-15	1	2
GM-CSF	0	2	IL-17	1	2
IL-1β	0	2	TNF-α	1	2
IL-2	0	2	IL-3	1	1
IL-6	0	2	MCP-2	2	3
IL-8	0	2	M-CSF	2	3
IL-11	0	2	TGF-β1	2	3
MCP-1	0	2	IL-7	2	2
TNF-β	0	2	MIP-1δ	2	1
ICAM-1	0	0	IFN-γ	3	4
IL-6sR	0	0	MIG	3	3
MIP-1β	0	0	IL-1α	3	2
RANTES	0	0	IL-13	3	2
sTNF RII	0	0	PDGF-BB	4	2

Levels of 40 different cytokines and chemokines associated with inflammatory responses were analyzed in plasma samples obtained from 4 OMT and 4 sham participants using a cytokine antibody array following the manufacturer's instructions. Plasma samples (pretreatment and 30 min post-treatment) from OMT and control participants were compared in order to determine changes in the levels of immune molecules due to treatment.

### Early response to OMT

**Figure 4:**
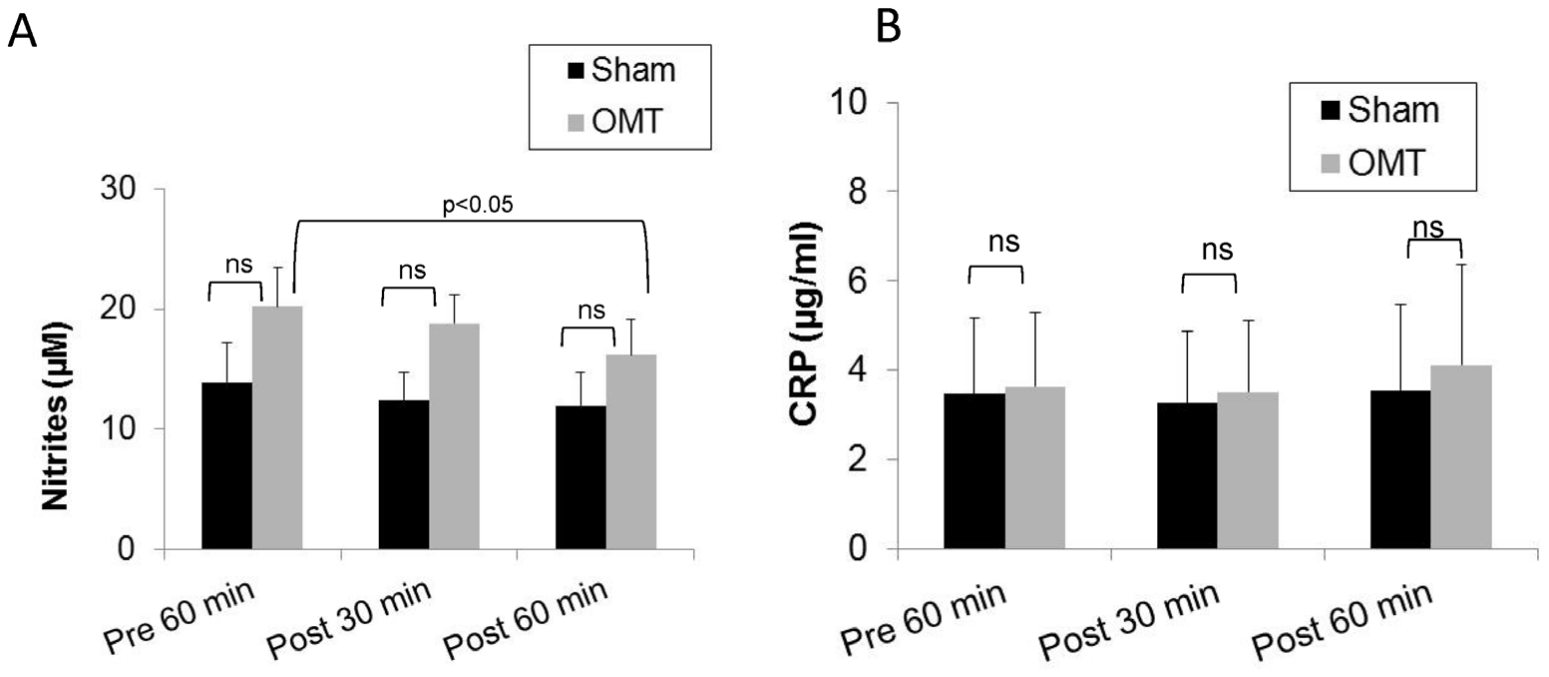
Levels of nitrites and CRP in plasma of OMT and sham experimental groups at 30 and 60-treatment. Plasma was recovered from blood samples and analyzed for the presence of nitrites (A) and CRP (B) as described in the Materials and Methods section. Data are expressed as mean ± SD. Data were analyzed by ANOVA followed by Tukey-Kramer Multiple Comparisons post-test. N = 16 OMT and 17 sham.

**Figure 5:**
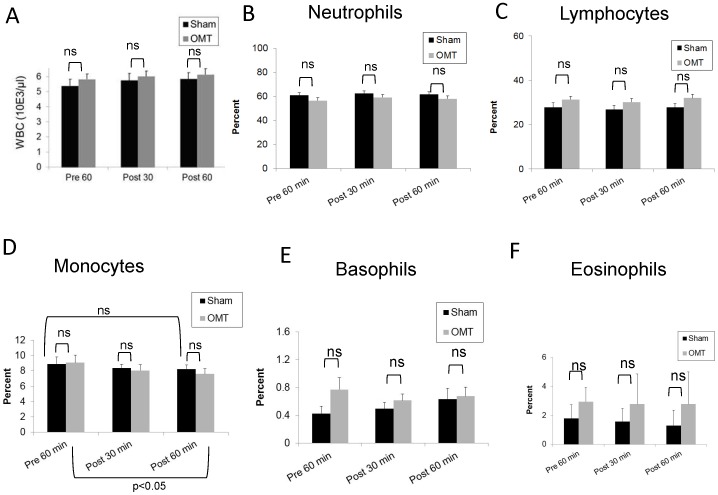
Total and differential WBC counts in OMT and sham experimental groups at 30 and 60-treatment. The total and differential numbers of white blood cells were determined in an automatic cell counter in triplicate (HemaVet HV950FS programmed for human blood). Data are expressed as mean ± SD. Data were analyzed by ANOVA followed by Tukey-Kramer Multiple Comparisons post-test. N = 16 OMT and 17 sham.

Taking into account this qualitative modification in the levels of some circulating cytokines without affecting leukocyte populations, we decided to change our time points for blood collection after OMT, selecting 30 min and 60 min post-treatment. We hypothesize that this would encompass the possibility of a modification in leukocyte levels in response to changes in the cytokine profile. As shown in **Fig. 4A**, a small but significant decrease in the levels of nitric oxide is observed only in the OMT group at 1 h post-treatment when compared with the values obtained at pre-treatment. As above, no differences were observed in the levels of CRP between OMT and sham-treated groups, or in the OMT group with respect to pretreatment levels (**Fig. 4B**). Similarly, as shown in **Fig. 5**, the total number and differential counts of leukocytes in blood were not modified with the exception of the monocyte population (**Fig. 5D**). Here, we observed a trend to a decrease in the level of monocytes by 1 h post-treatment in sham and OMT groups, that was significant for the OMT group (p<0.05). Thus, it is tempting to speculate that the small, but significant decrease in the levels of nitrites in circulation could be related to a higher decrease in the monocytes values, taking into account that these cells constitutively express nitric oxide synthases [33]. It is noteworthy to comment that in our hands, three successive venipuncture procedures had an effect by itself on the proportion of circulating antigen presenting cells, which becomes more evident by flow cytometry analysis as described below.

**Figure 6:**
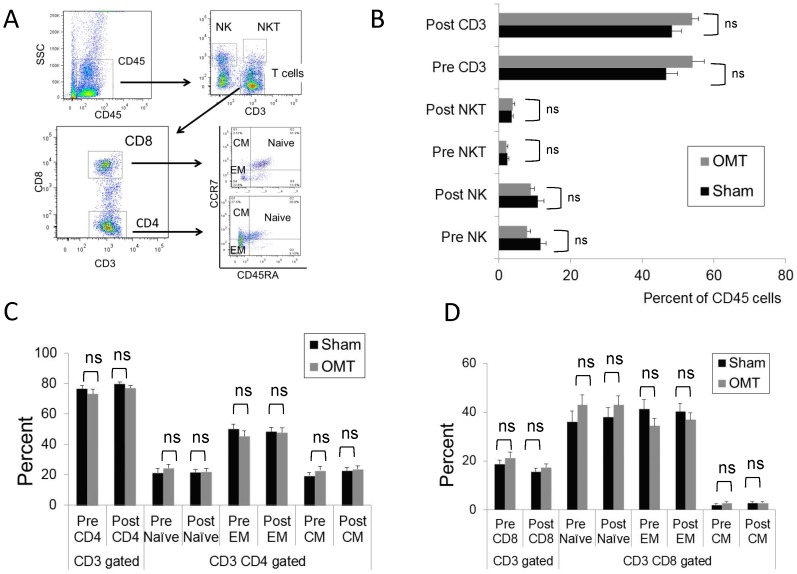
Flow cytometry analysis of circulating NK, NKT and T cell populations in OMT and sham experimental groups at 60-treatment. (**A**) Gating strategy to define NK, NKT and T cell percentages within the CD45 fraction of PBMC. (**B**) Percentage of CD3, NK and NKT cells within the CD45 PBMC fraction. Distribution of different CD4 (B) and CD8 (**C**) populations within the CD3/CD45 fraction of PBMCs. Data are expressed as mean ± SD. Data were analyzed by ANOVA followed by Tukey-Kramer Multiple Comparisons post-test. N = 16 OMT and 17 sham.

**Figure 7:**
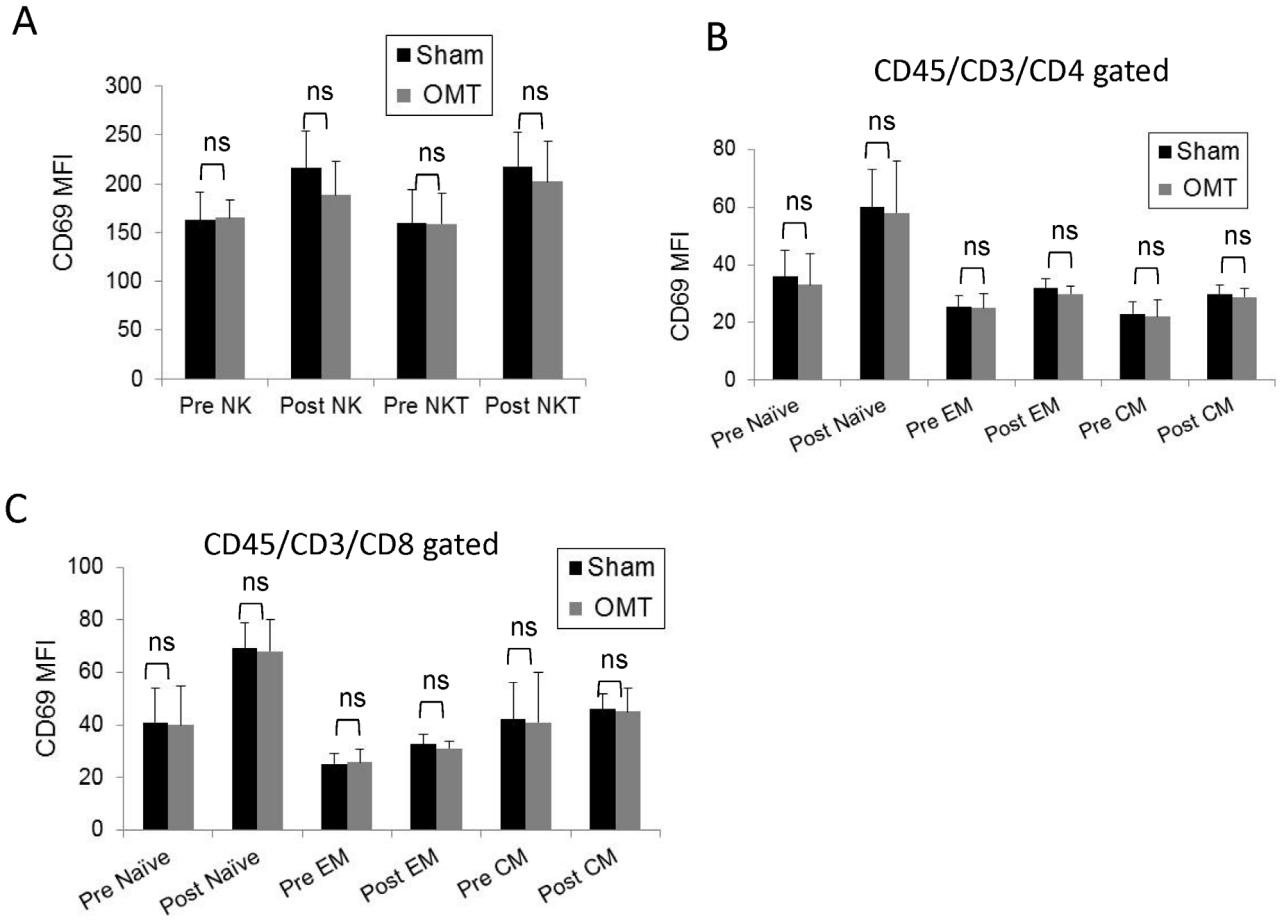
Activation status of circulating NK, NKT and T cell populations in OMT and sham experimental groups at 60-treatment. Mean fluorescence intensity (MFI) values of CD69, an early activation antigen, were determined in NK and NKT cells (**A**), and Central and effector memory and naïve CD (**B**) and CD8 (**C**) T cells. Data are expressed as mean ± SD. Data were analyzed by ANOVA followed by Tukey-Kramer Multiple Comparisons post-test. N = 16 OMT and 17 sham.

**Figure 8:**
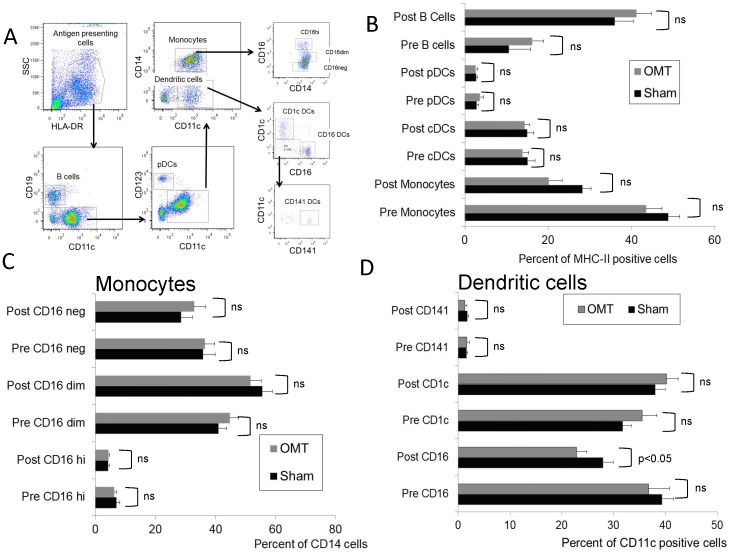
Flow cytometry analysis of circulating APC populations in OMT and sham experimental groups at 60-treatment. B cells and different subpopulations of monocytes and dendritic cells were analyzed within the HLA-DR fraction of PBMCs. (**A**) Gating strategy to define antigen presenting cells. (**B**) Percentage of B cells, monocytes and DCs within the HLA-DR fraction. Distribution of different monocyte (**C**) and dendritic cell (**D**) populations within the APC fraction of the PBMCs. Percentages of the different populations were determined by comparison with isotype controls Data are expressed as mean ± SD. Data were analyzed by ANOVA followed by Tukey-Kramer Multiple Comparisons post-test. N = 16 OMT and 17 sham.

We then performed an exhaustive flow cytometry analysis of PBMCs recovered from OMT and sham-treated groups 1 h post-treatment. **Fig. 6A** shows the gating strategy applied to the PBMC population to investigate NK, NKT, and T cells (central memory, effector memory and naïve CD4 and CD8 cells). As shown in **Fig. 6B**–**D**, no differences were observed in these populations, or their activation status as determined by evaluation of the CD69 expression (**Fig. 7**). In a complementary series of studies, we decided to investigate the antigen presenting cell (APC) population within the PBMC fraction recovered from OMT and sham groups at 1 h post-treatment. **Fig. 8A** shows the gating strategy to analyze APCs as HLA-DR (MHC-II) positive cells, expressing diverse surface antigens such as CD19 (B cells), CD14 (monocytes) and CD11c or CD123 (conventional and plasmacytoid DCs respectively). Further, different populations of monocytes and DCs were identified by using previously described markers [34], [35]. As above, 3 successive venipuncture procedures had an effect on the proportion of PBMC populations. Most notably, we were able to detect significant increases in the proportions of B cells in this fraction together with a significant decrease in the proportion of monocytes in both OMT and sham groups. It needs to be highlighted that this analysis was performed on the PBMC fraction of the blood in contrast with the above data on monocytes and lymphocytes that was performed on whole blood. Interestingly, the trend in the decrease in the levels of monocytes was more pronounced in the OMT group (**Fig. 8B**). More important, we were able to observe a decrease in the levels of a subpopulation of DCs. As shown in **Fig. 8D**, the CD16^+^ DC subpopulation was present at significantly lower levels in the PBMC fraction recovered from the OMT group. The significance of this observation is very important. DCs are the most powerful APCs present in the body, and as such have the capability to stimulate specific T cell responses [36], [37], [38]. For example, these cells are responsible for the success of a vaccination procedure and as such have been considered as a platform for cell based vaccines [39], [40]. In particular, the CD16^+^CD11c^+^HLA-DR^+^ population has been described as a subset of DCs involved in inflammatory responses, since they are characterized by high expression of TNFα (and low levels of IL-10) upon stimulation [34], [41]. We interpreted these data as indicative of a modification in the biodistribution of a particular subpopulation of DCs induced by OMT. It is tempting to speculate that OMT, acting as a mechanotransduction stimulus, induces some DCs to persist at the level of lymph nodes, or accelerate their migration to the tissues. This finding can help interpret historical data claiming a role of OMT on improving the body defenses as described above. As recently reviewed by Hodge (2012), OMT has the capability to act as an adjunctive therapy to improve cleansing of the tracheobronchial tree, increase sputum production and shorten the duration of coughing in patients with pneumonia [42]. This could potentially increase the availability of the pathogen to the immune system that when combined with increased mobilization of DCs might help fight an infection.

**Figure 9:**
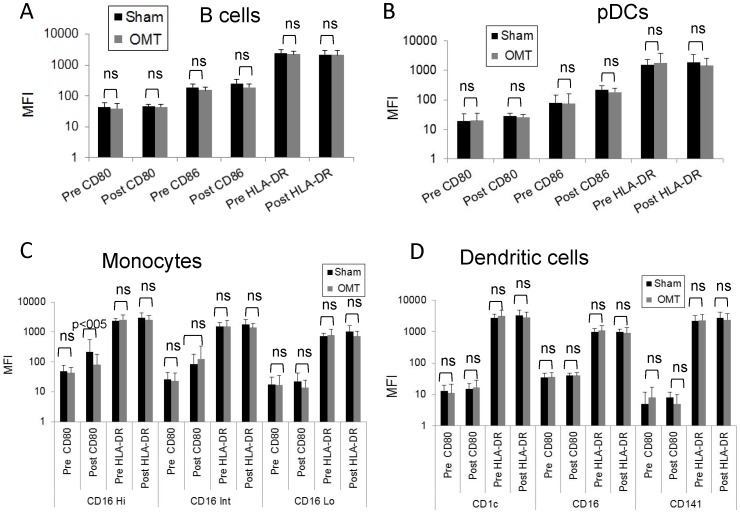
Activation status of circulating APC populations in OMT and sham experimental groups at 60-treatment. MFI values of CD80, CD86 and HLA-DR were determined in B cells (**A**) and plasmacytoid DCs (**B**) present in the PBMC fraction of OMT and sham-treated groups 1 h after treatment. MFI values of CD80 and HLA-DR were determined in monocyte (**C**) and dendritic cell subpopulations (**D**) present in the PBMC fraction of OMT and sham-treated groups 1 h after treatment. Data are expressed as mean ± SD. Data were analyzed by ANOVA followed by Tukey-Kramer Multiple Comparisons post-test. N = 16 OMT and 17 sham.

When we analyzed APCs activation markers (i.e., the levels of expression of costimulatory molecules CD80, CD86 and HLA-DR), no differences in the activation levels of B cells, DCs or plasmacytoid DCs were observed in our samples upon OMT (**Fig. 9A, B and D**). We were able to determine a significant difference in the levels of CD80 in CD16^hi^ monocytes between OMT and sham groups (**Fig. 9C**). It is noteworthy to comment that successive blood draws increased the levels of this population in circulation in the sham group as shown when compared pre and post-treatment, and our interpretation is that in the sham group, cells with lower levels of CD80 expression, presumably more immature forms, rapidly abandon circulation. Our rationale is that during the length of our study there was not enough time to induce higher expression of this molecule by de novo synthesis, thus any modification in the observed levels should be due to mobilization of particular subpopulations. The difference in this behavior between OMT and sham samples further supports the possibility of OMT modifying immune parameters.

**Figure 10:**
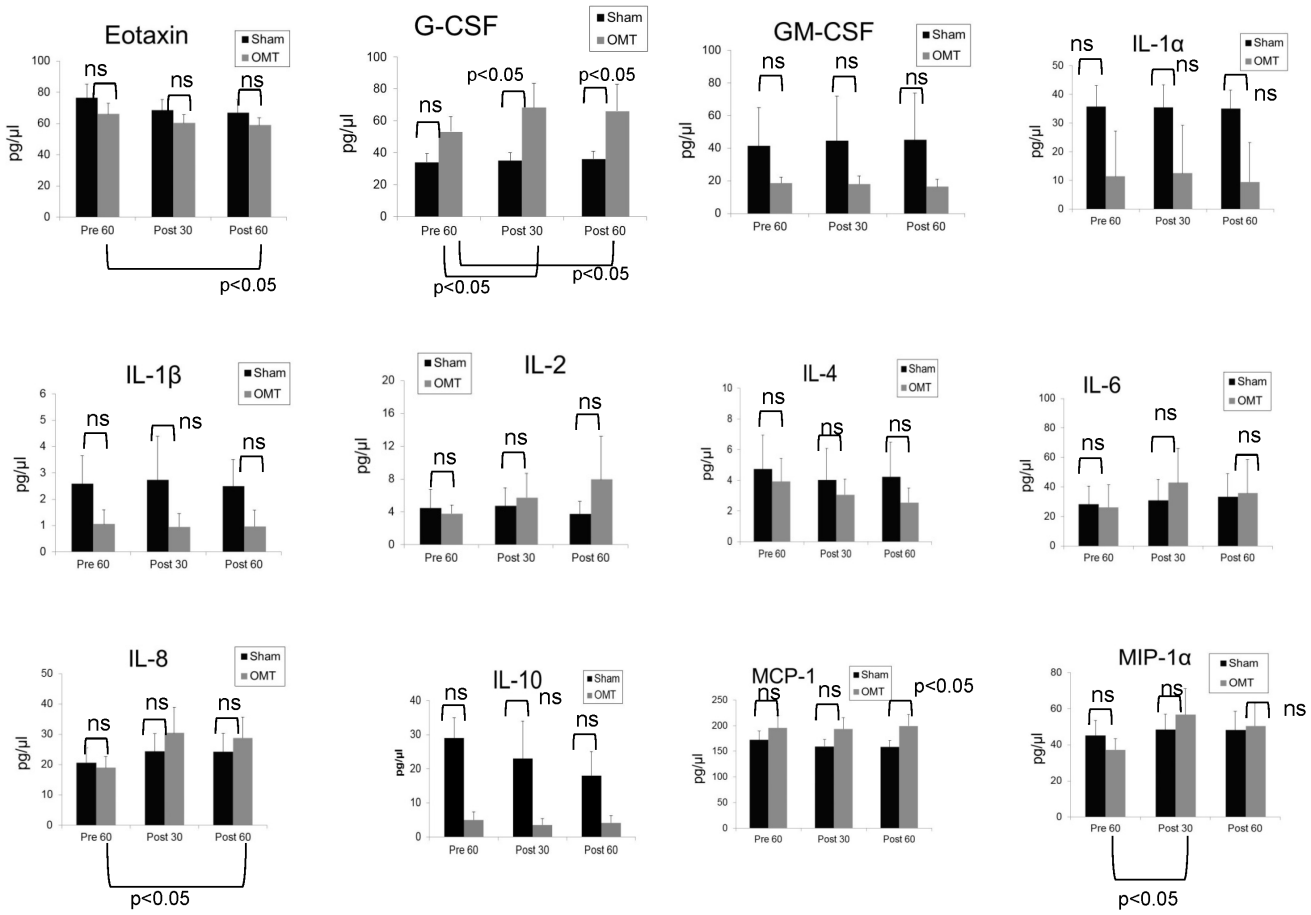
Multiplex analysis of plasma chemokine and cytokine populations in OMT and sham experimental groups at 30 and 60-treatment. The levels of specific chemokines, cytokines or growth factors were determined by using a custom Milliplex Map Kit human cytokine panel. Data are expressed as mean ± SD. Data were analyzed by ANOVA followed by Tukey-Kramer Multiple Comparisons post-test. When samples did not follow normal distribution, non-parametric statistics were used for analysis. When comparing the levels of a particular molecule within the OMT or sham group at different time-points, we used paired-sample statistics analysis. Overall, the statistical analysis was focused on each specific cytokine expressed by supernatants recovered from different environments, but no comparison was performed between different cytokines. N = 12 OMT and 14 sham.

Finally, in a series of complementary studies we investigated the effect of OMT on the circulating levels of cytokines that were modified in our first series of qualitative studies. As shown in **Fig. 10**, significant variations in the levels of specific cytokines are observed. Firstly, no differences in the plasma levels of eotaxin, GM-CSF, IL-1α, IL-1β, IL-2, IL-4, IL-6, and IL-12 and were observed upon OMT. On the other hand, we were able to detect a significant increase in the plasma levels of MIP-1α in the OMT group compared with pre-treatment values, 30 min post-treatment. This was not observed in the sham group. Similarly, a significant increase in plasma levels of IL-8 was observed in the OMT group compared to basal values 60 min after treatment. In addition, the levels of MCP-1 were also significantly higher in the OMT group compared to sham controls at 60 min post-treatment, mostly due to a decrease in the levels of this cytokine in normal controls. This again draws attention to the effect of repeated venipuncture on hematological parameters.

More importantly, plasma levels of G-CSF, an inducer of monocyte production by the bone marrow, were significantly upregulated only in the OMT group both at 30 and 60 min post-treatment. This further highlights the effects of OMT on the monocyte-dendritic population, since the CD16 DC population is considered to be derived from CD16 monocytes, and shares characteristics with them. In all, some of this cytokine data is in line what we have observed in our first series of experiments and further strengthen our data pointing towards an immunomodulatory role of OMT.

### Limitations of the study

The present study has several limitations. First, as described in related studies [28], the small sample size means only large between-group differences were likely to be detected. Thus, differences in blood lymphocytes, basophils and eosinophils 60 min upon OMT may have been significant. On the other hand, a retrospective statistical analysis, confirmed that the significant differences observed in the levels CD16 DCs and in the cytokines and chemokines concentrations determined through multiplex analysis, retained a statistical power of 0∶8.

In addition, another limitation of these studies associated to a small sample size is that although demographic data was collected, we were unable to perform a stratified analysis of our results to include gender and age differences. A study involving a larger population would be able to identify differential effects of OMT associated to age and/or gender.

Finally, an additional limitation of our studies is that only short-term effects of OMT (1 h after treatment) were evaluated. Long-term effects involving *de no*vo synthesis of cytokines or chemokines were not investigated here and future studies should include them in order to fully understand the effect of OMT on immune parameters. In this context, it is noteworthy to highlight that we included only healthy individuals in these studies. A study involving individuals suffering inflammatory conditions in which several chemokines and cytokines are being produced at higher levels than normal by activated cells, could help identify the effect of OMT on the regulation of cytokine, chemokine or immune-related growth factor production. Indeed, studies on the effect of OMT on elderly populations [28], which are considered to have low levels of chronic inflammation, might be able to address this issue.

## Conclusion

In closing, our data show for the first time that OMT exerts a modification in the distribution of a particular blood DC population. This was possible due to thorough flow cytometry analysis and multiplex technology. This could help interpret data indicating that OMT can help fight infections, or can increase the efficacy of vaccinations. Furthermore, this argues for extended immunological studies aiming to determine that OMT might help some conventional anti-infectious therapies. In this context, combination of OMT with conventional therapeutic treatments has the potential of lowering healthcare costs which will result in a great benefit for both the patient and the healthcare system. Finally, this study focused in changes induced by OMT in which little or no effect from *de novo* synthesis of immune molecules could be surely observed due to the limited time lapse between treatment and sampling. Even under these stringent conditions we were able to detect some modification in immune parameters in our studied population. This argues for future studies investigating the effect of OMT at later time points in order to observe effects on the synthesis of circulating immune molecules or the upregulation of activation markers in immune cells.

## Supporting Information

File S1 **Patient consent form regarding the studies described herewith.** Each participant read and signed the consent form document.
(DOC)
