# Supplementary material for: Osteopathic Manipulative Therapy Induces Early Plasma Cytokine Release and Mobilization of a Population of Blood Dendritic Cells
Source: PLoS One. 2014 Mar 10;9(3):e90132. doi: 10.1371/journal.pone.0090132 (PMC3948629; doi:10.1371/journal.pone.0090132)
Supplement: File S1 — Patient consent form regarding the studies described herewith. Each participant read and signed the consent form document. (DOC) [file pone.0090132.s001.doc]

Ohio University Consent Form

Title of Research: MECHANISMS OF IMMUNE MODULATION BY OSTEOPATHIC MANIPULATIVE TREATMENT-INDUCED CYTOKINE RELEASE

Researchers: Fabian Benencia, Ph.D., Kenneth J. Goodrum, Ph.D., Stevan Walkowski, D.O.

You are being asked to participate in research. For you to be able to decide whether you want to participate in this project, you should understand what the project is about, as well as the possible risks and benefits in order to make an informed decision. This process is known as informed consent. This form describes the purpose, procedures, possible benefits, and risks. It also explains how your personal information will be used and protected. Once you have read this form and your questions about the study are answered, you will be asked to sign it. This will allow your participation in this study. You should receive a copy of this document to take with you.

# Explanation of Study

Research focused on the mechanism whereby osteopathic manipulative treatment (OMT) may enhance the body’s natural resistance to infection is being conducted. OMT is a form of manual therapy that uses physical contact to improve the impaired or altered function of the musculo-skeletal system. OMT also improves resistance to infection by increasing the movement and contact of tissue fluids (lymphatic fluid) with tissues of the immune system. You will be randomly assigned into one of two groups and will receive one of two types of OMT for a total of about 7 minutes by a licensed osteopathic physician. OMT will involve placement of the physician’s hands on your chest, abdominal area, and back with or without repeated manual compression of these areas. One hour prior to OMT and at 30 minutes and 60 minutes after OMT, blood samples will be collected from your arm vein (venipuncture of median cubital vein).There will be three (3) venipunctures with a total of 72 ml or 1/3 of a measuring cup of blood drawn from your arm. This is 1/6 of the amount usually donated at the Red Cross. Blood (cells and serum) from the different time points will be tested in a laboratory for changes in immune cell numbers and functions associated with natural resistance to infection. This single OMT treatment and 3 donations of blood is your sole participation in this research.

To participate in this study you must be between 18 and 65 years old; must be a nonsmoker; must not use recreational drugs; must not be pregnant; must not have been treated (within past 3 months) with corticosteroids, cytotoxic drugs, or immunosuppressants; must not have liver disease, renal failure, acute and chronic infections (including HIV); must not have recently broken or damaged ribs or upper body injury (within past 6 weeks); must not have been told within the last 8 weeks that you are anemic; must not have donated 500cc or more blood in the last 8 weeks; must not get short of breath while walking or get lightheaded when going from lying down to sitting, or sitting to standing; must not have used anti-inflammatory medications or consumed alcohol for one week prior to your participation.

## Risks and Discomforts

Risk and discomfort with OMT is minimal and unlikely, but may include headache, flu-like symptoms, or musculo-skeletal aches lasting 1-2 days after treatment. Also, with the exception of augmented breathing on occasion, the treatments will not require active participation by you. Possible risks and discomforts from drawing blood include bleeding, hematoma (a localized, confined mass of blood which has leaked into tissues surrounding the site where the needle has punctured the vein. The blood may clot leaving a temporary lump or discoloration under the skin.), pain, and bruising at the blood drawing site. These risks are low and the discomfort is temporary and minor. At no time will another person’s blood come into contact with yours. No alternative procedures are available. In the unlikely event of physical injury, immediate medical treatment is available at O’Bleness Memorial Hospital.

## Benefits

Benefits of participation include education about possible benefits of OMT and knowledge that the use of your blood in this study could promote more effective patient care via OMT. Because ill patients will be excluded from the study, it is not likely that you will experience a symptomatic benefit. If these studies identify and explain a beneficial effect of OMT on natural resistance to infection, society would benefit by broader societal acceptance of OMT as an alternative therapy in patient populations with declining immunity.

## Confidentiality and Records

The 3 blood donations and OMT is your only participation in this project and your name will in no way be connected or recorded in relation to any of the results. Your medical history information will be collected solely for determination of exclusion criteria and will not be used for research purposes. At the time of enrollment, you will be identified by a study code number that will be used to track study results.  Your blood samples will be identified only by study code number. The study code key will be stored in a locked cabinet in the office of Stevan Walkowski, D.O. Any personal identifying information on you will be kept in a locked cabinet at the office of Lynn Petrik, R.N.. The identifying information will be destroyed within 90 days after the conclusion of the study.  Your name, address, and social security number will be provided to the Ohio University Finance office for you to receive compensation.

Additionally, while every effort will be made to keep your study-related information confidential, there may be circumstances where this information must be shared with:

* Federal agencies, for example the Office of Human Research Protections, whose responsibility is to protect human subjects in research;

* Representatives of Ohio University (OU), including the Institutional Review Board, a committee that oversees the research at OU;

**Compensation**

You will receive $40 for participation in this study even if you choose not to complete your participation.

## Contact Information

If you have any questions regarding this study or to report a research-related injury, please contact Kenneth J. Goodrum, Ph.D., [goodrum@ohio.edu](mailto:goodrum@ohio.edu), 740-593-2390 or Stevan Walkowski, DO, 740-593-2231, [walkowsk@ohio.edu](mailto:walkowsk@ohio.edu)

If you have any questions regarding your rights as a research participant, please contact Jo Ellen Sherow, Director of Research Compliance, Ohio University, (740)593-0664.

By signing below, you are agreeing that:

- you have read this consent form (or it has been read to you) and have been given the opportunity to ask questions
- known risks to you have been explained to your satisfaction.
- you understand Ohio University has no policy or plan to pay for any injuries you might receive as a result of participating in this research protocol
- you are 18 years of age or older and meet study subject selection criteria.
- your participation in this research is given voluntarily
- you may change your mind and stop participation at any time without penalty or loss of any benefits to which you may otherwise be entitled.

Signature Date

Printed Name

Name and signature of person obtaining the informed consent:

Printed name ________________________________

Signature _______________________________________ Date _____________
